# Selection and T-cell antigenicity of synthetic long peptides derived from SARS-CoV-2

**DOI:** 10.1099/jgv.0.001698

**Published:** 2022-01-11

**Authors:** Katarzyna Piadel, Amin Haybatollahi, Angus George Dalgleish, Peter Lawrence Smith

**Affiliations:** ^1^​ Institute of Infection and Immunity, St Georges University of London, London UK

**Keywords:** SARS-CoV-2, T-cells, peptide, vaccine

## Abstract

The pandemic caused by SARS-CoV-2 has led to the successful development of effective vaccines however the prospect of variants of SARS-CoV-2 and future coronavirus outbreaks necessitates the investigation of other vaccine strategies capable of broadening vaccine mediated T-cell responses and potentially providing cross-immunity. In this study the SARS-CoV-2 proteome was assessed for clusters of immunogenic epitopes restricted to diverse human leucocyte antigen. These regions were then assessed for their conservation amongst other coronaviruses representative of different alpha and beta coronavirus genera. Sixteen highly conserved peptides containing numerous HLA class I and II restricted epitopes were synthesized from these regions and assessed *in vitro* for their antigenicity against T-cells from individuals with previous SARS-CoV-2 infection. Monocyte derived dendritic cells were generated from these peripheral blood mononuclear cells (PBMC), loaded with SARS-CoV-2 peptides, and used to induce autologous CD4+ and CD8+ T cell activation. The SARS-CoV-2 peptides demonstrated antigenicity against the T-cells from individuals with previous SARS-CoV-2 infection indicating that this approach holds promise as a method to activate anti-SAR-CoV-2 T-cell responses from conserved regions of the virus which are not included in vaccines utilising the Spike protein.

## Data Summary

NC_045512.2, MN996532.2, MT072864.1, MT040333.1, KC881005.1, KY417144.1, KT444582.1, KF367457.1, KC881006.1, KY417152.1, MK211376.1, AY572034.1, AY572035.1, AY572038.1, AY686864.1, AY686863.1, NC_004718.3, AY390556.1, KY352407.1, NC_014470.1, GU190215.1, MG772933.1, MG772934.1, MK211374.1, JX993987.1, MK211377.1, DQ084199.1, GQ153539.1, DQ084200.1, DQ022305.2, KJ473815.1, KJ473813.1, KJ473812.1, KP886808.1, DQ648857.1, DQ412043.1, KY417147.1, DQ648856.1, JX993988.1, FJ588686.1, MK211378.1, KJ473816.1, KJ473811.1, MG596802.1, MG596803.1, NC_009020.1, MG021452.1, MK129253.1, JX869059.2, MT576585.1, NC_038294.1, DQ648794.1, KJ473821.1, KJ473822.1, KX442564.1, EF065505.1, NC_009019.1, KC869678.4, NC_039207.1, KC545386.1, KJ473806.1, NC_026011.1, NC_017083.1, FJ647223.1, KF686343.1, AY391777.1, NC_030886.1, NC_009021.1, HM211098.1, MK211375.1, KY770860.1, KU182964.1, MT430884.1, LC469308.1, NC_048212.1, MF593268.1, NC_034440.1, NC_028814.1, JQ989273.1, NC_018871.1, AF304460.1, JQ410000.1, NC_048216.1, AY567487.2, NC_046964.1, NC_028833.1, KJ473820.1, NC_028811.1, NC_022103.1, EF203064.1, NC_028824.1, DQ648858.1, EU420138.1, KJ473795.1, EU420137.1.

## Introduction

The pandemic caused by the novel coronavirus, SARS-CoV-2, has prompted a global response to produce effective vaccines. A number of vaccines have been approved for use having demonstrated varying levels of efficacy in clinical trials [1]. These vaccines, often based upon targeting the viral Spike protein, responsible for facilitating entry of SARS-CoV-2 into cells [2], have demonstrated an ability to limit infection, transmission and the onset of serious disease. However, a number of issues remain. The recent identification of SARS-CoV-2 variants to which vaccine elicited immune protection may be reduced [3] raises the prospect of continued susceptibility to serious infection and the need for repeated vaccination to raise immunity to new variants or novel coronavirus outbreaks. Additional problems related to vaccination include cost, logistics and the duration of protection afforded by neutralising antibody [4].

T-cell responses to SARS-CoV-1 demonstrate greater durability than those of neutralising antibody [5] and are associated with protection against SARS-CoV-2 [6], particularly in the context of waning antibody titres [7], indicating that T-cell mediated immunity may offer durable immune protection which may limit the severity of disease and potentially offer immune responses that are cross reactive to variant SARS-CoV-2 and other coronaviruses [6, 8, 9] similar to those have observed for different influenza viruses [10]. T-cell immune responses are generated by vaccination with the SARS-CoV-2 spike protein however these responses are thought to represent approximately 50% of the total anti-SARS-CoV-2 CD4+ T cell response and 25% of CD8+ T cell responses [11]. Therefore, spike-based vaccines will likely induce sub-optimal anti-SARS-CoV-2 T-cell responses and alternative methods of inducing T-cell immunity need to be explored. Here we provide a rationale for the selection of antigenic regions from SARS-CoV-2 proteins including the nucleoprotein, membrane protein, envelope protein, ORF3, ORF7a and the non-structural proteins intended to provide broad T-cell activation and assess these synthetic long peptides for immunogenicity *in vitro*.

## Methods

### Coronavirus sequence conservation

Analysis of SARS-CoV-2 and other coronaviruses protein sequences from proteins harbouring epitope rich regions was performed using FASTA sequences deposited in the NCBI database (https://www.ncbi.nlm.nih.gov/labs/virus/vssi/), alignment using Clustal Omega with default settings (https://www.ebi.ac.uk/Tools/msa/clustalo/) and analysis of conservation using Microsoft Excel. Accession numbers of coronaviruses strains can be found in the supplementary material file.

### *In silico* prediction of T-cell epitopes

Identification of SARS-CoV-2 HLA restricted epitopes using prediction algorithms and experimentally validated epitopes deposited in the Immune epitope database and by using the NetMHCpan EL 4.1 (2020.09) prediction algorithm against an HLA allele reference set (https://www.iedb.org/).

### Selection and synthesis of synthetic long peptides

Selection of conserved immunogenic regions between 15–30 amino acids in length (synthetic long peptides) was determined by assessing their suitability for synthesis based upon the physiochemical properties of the amino acids in the sequence and potential as CPP (cell penetrating peptides) defined by a net positive charge. Selected peptides were synthesized (Genscript, Netherlands).

### Peripheral blood mononuclear cells

Peripheral blood mononuclear cells (PBMC) were purchased from the national blood service (NHS, UK) prior to the distribution of SARS-CoV-2 vaccines. PBMC demonstrating responses to a SARS-CoV-2 consensus peptide pool and serum antibody responses to the SARS-CoV-2 spike protein were defined as having been previously infected with SARS-CoV-2 whilst PBMC and sera lacking detectable responses were defined as SARS-CoV-2 naïve.

### Isolation of PBMC and generation of monocyte-derived DC

The generation of mDDC (monocyte-derived dendritic cells) was performed using established protocols. CD14+ cells were isolated by positive selection using anti-CD14 conjugated magnetic beads (Miltenyi, Germany). The CD14+ cells were cultured for 6 days in RPMI (Sigma, UK) 10% foetal calf serum (sigma, UK) and 5% streptomycin/penicillin (Sigma, UK) of 10 ng ml^−1^ IL-4 and 50 ng ml^−1^ GM-CSF (Miltenyi, Germany). These mDDC were subsequently co-cultured with the T-cells enriched from the CD14- fraction of PBMC using anti-CD3 magnetic beads (Miltenyi, Germany) in the presence of SARS-CoV-2 peptides individual and in groups, and control peptides including CEF (Cytomegalovirus/Epstein Barr virus and influenza), CEFT (Cytomegalovirus/Epstein Barr virus/influenza/tetanus) SARS-CoV-2 reference group (including overlapping peptides from the spike, nucleocapsid and membrane) (1 µg ml^−1^ for each peptide) for antigenicity assays.

### ELISPOT

IFN-γ ELISPOT (enzyme-linked immunosorbent spot) assays using peptide pulsed mDDC-T-cell co-cultures (2×10^4^ mDDC: 2×10^5^ T-cells) were incubated in IFN-γ ELISPOT plates (CTL ltd, Germany) for 48 h in order to assess antigen specific T-cell activation. After 48 h ELISPOT plates were processed according to the manufacturers protocol. Briefly, plates were washed in PBS ×3 prior to the incubation with detection anti-IFN-γ antibody for 2 h. Plates were washed ×3 in PBS wash buffer and Strep-Biotin reagent was added for 1 h. Plates were washed ×3 prior to the addition of substrate solution. Spot formation was observed and the plates were washed once in distilled H_2_O and left to dry before enumeration using a CTL Immunospot entry ELISPOT plate reader. Positive responses compared to the no peptide control were defined as >1.5-fold change and statistical significance using Student’s t-test.

### Flow cytometry

Multiparameter flow cytometry was used to measure CD8+ and CD4+ T cell activation using the AIM assay. A total of 1×10^5^ MDDC were incubated with SARS-CoV-2 or control peptides and co cultured with 1×10^6^ of the T-cells enriched from the CD14- fraction of PBMC for 24 h in flat bottomed 96 well plates prior to staining. V450 anti-CD3, AF488 anti-CD8, APC-fire-anti-CD4, V670 anti-CD45RA and V710 anti-CCR7 were used as T-cell subset markers. APC anti-CD69, Percp anti-CD137 and PE-dazzle anti-OX40, Alexa Fluor 700-OX were used as activation induced markers. Responses to the peptides were defined as a 1.5-fold increase to the no peptide control.

## Results

### Identification of antigenic regions within the SARS-CoV-2 proteome

In order to identify amino acid sequences within the SARS-CoV-2 proteome that contain multiple class I and II restricted epitopes, peptides from conserved regions from SARS-CoV-2 proteins were assessed using the IEBD epitope prediction tool [12]. Identification of clusters of epitopes, previously validated for HLA binding or T-cell activation and deposited within the IEDB database, were also used. This resulted in the identification of 25 peptide regions harbouring multiple predicted or experimentally validated epitopes. Five of these peptides were identified within the Spike protein and were not investigated further since T-cell responses to these regions may be raised by existing vaccines. The remaining 20 peptides were derived from the nucleoprotein, envelop protein, membrane protein, ORF3a, ORF7a and the ORF1ab polyprotein (Table 1). These peptides were assessed for conservation between 500–2500 SARS-CoV-2 protein sequences deposited on the NCBI virus database and including sequences derived from different geographic locations and belonging to variants of concern. The amino acids in each peptide were highly conserved with typically between 98–100% conservation for each aa residue within each peptide (Table S1). Some peptides demonstrated 100% conservation whilst the average conservation across all 20 peptides was 99.4 % (Table 1). The limited variation was often between similar amino acids (Table S1). Early analysis of available protein seqeunces from the Omicron varient of SARS-CoV-2 also demonstrated 99% conservation with the peptides identified here.

**Table 1.** Immunogenic SARS-CoV-2 peptides. The peptides were identified based upon the presence of HLA class I and II restricted epitopes, sequence conservation amongst SARS coronaviruses, suitability for synthesis and positive charge at neutral pH

No	Region	Sequence	Sequence length	Hydrophobicity	Gravy	MW average G mol^−1^	Theoretical pI	Average aa conservation (%)
**1**	NP^41-57^	RRPQGLPNNTASWFTALTQHGKEDLK	26	50.44	−0.88	2864.2423	10.1	99.0
**2**	NP^65-94^	FPRGQGVPINSGPDDQIGYYRRATRRI	27	31.61	−1.04	3090.4843	11.1	99.3
**3**	NP^100-28^	KMKDLSPRWYFYYLGTGPEAGLPYGANK	28	43.16	−0.75	3223.7303	9.7	99.4
**4**	NP^215-229^	GDAALALLLLDRLNQLESKMSGK	23	44.86	−0.29	2769.2873	10.6	99.3
**5**	NP^311-335^	SASAFFGMSRIGMEVTPSGTWLTYT	25	46.39	0.26	2698.0933	6.9	98.9
**6**	NP^350-374^	VILLNKHIDAYKTFPPTEPKKDKKK	25	33.36	−1.06	2952.5603	10.3	99.2
**7**	M^94-111^	SYFIASFRLFARTRSMWSFNPETNIL	26	59.53	0.03	3155.6593	11.1	100.0
**8**	M^136-158^	SELVIGAVILRGHLRIAGHHLGR	23	41.21	0.51	2474.9643	12.2	100.0
**9**	E^50-69^	SLVKPSFYVYSRVKNLNSSRV	21	36.83	−0.23	2443.8583	10.8	100.0
**10**	ORF3^202-19^	VLHSYFTSDYYQLYSTQLRR	20	41.13	−0.67	2540.8443	9.4	99.9
**11**	ORF7a^76-97^	QLRARSVSPKLFIRQEEVQELYR	23	37.01	−0.8	2846.3133	10.4	97.3
**12**	NSP3^2323-40^	RRLVAEWFLAYILFTRFFYVRR	22	73.42	0.15	2987.5793	11.2	98.2
**13**	NSP6^3622-647^	SAFAMMFLVKHKHAFFLLPSLATVA	25	65.11	1.22	2854.5453	10.6	98.2
**14**	NSP8^4082-102^	RRNTCDGNTFTYASALWEIQQVVRR	25	45	−0.76	2985.3633	9.9	99.3
**15**	NSP12^4894-915^	DALFAYTKRNVIPTITQMNLKY	22	42.89	−0.16	2601.0893	10	99.9
**16**	NSP12^5246-63^	LMIERFYSLAIDAYPLTKRR	20	49.67	0.2	2392.9163	10.4	100.0
**17**	NSP12^5267-95^	QEYADVFHLYLQYIRKRLTGHMLDMYR	27	53.9	−0.57	3461.0613	9.3	100.0
**18**	NSP13^5455-79^	KLFAAETLKATEETFKLSYGIATVR	25	43.63	0.04	2788.2603	9.6	100.0
**19**	NSP13^5745-58^	RRCPAEIVDTVSALVY	18	35.09	−0.13	1792.1063	9.9	100.0
**20**	NSP16^6821-45^	KYTQLCQYLNTLTLAVPYNMRVIHF	25	53.14	0.16	3030.6383	9.5	100.0

Peptides intended to induce broad antigen specific T-cell responses need to contain epitopes to the most common HLA alleles in human populations. The collective HLA restriction of the experimentally validated epitopes identified in the IEDB database in each of the 20 peptides was determined (Table 2). The 20 peptides included a total of 144 experimentally validated epitopes, 125 restricted to HLA class I and 19 restricted to HLA-class II. Next the presence of predicted, but untested, epitopes was determined (Table 3) defined as being within the top 0.1% of predicted binders for each HLA allele. Ninety-five predicted epitopes were identified, 93 restricted to HLA class I and 2 to HLA-class II. In total the epitopes identified within the peptides were restricted to the most common HLA-class I alleles including but not limited to HLA*A01 : 01, HLA*A02 : 01, HLA*A03 : 01, HLA*A11 : 01, HLA*A24 : 02, HLA*A68 : 01, HLA*A68 : 02, HLA*B07 : 02, HLA*08 : 01, HLA-B*15 : 02, HLA*B35 : 01, HLA*B40 : 01.

**Table 2.** Experimentally proven SARS-CoV-2 epitopes present in the selected peptides. SARS-CoV-2 epitopes and their HLA restriction present in each peptide from epitopes deposited based upon T-cell activation data and/or MHC binding data deposited on the IEDB database

HLA	No	1	2	3	4	5	6	7	8	9	10	11	12	13	14	15	16	17	18	19
		NP^41-57^	NP^65-94^	NP^100-28^	NP^215-229^	NP^311-335^	NP^350-374^	M^94-111^	M^136-158^	E^50-69^	ORF3^202-19^	ORF7a^76-97^	NSP3^2323-40^	NSP6^3622-647^	NSP8^4082-102^	NSP12^4894-915^	NSP12^5246-63^	NSP13^5455-79^	NSP13^5745-58^	NSP16^6821-45^
A*01 : 01	5			DLSPRWYFY GTGPEAGLPY		VTPSGTWLTY					FTSDYYQLY				NTCDGNTFTY					
A*02 : 01	17			YLGTGPEASL	LALLLLDRL, RLNQLESKV	GMSRIGMEV	ILLNKHIDA, ILLNKHID	SMWSFNPETNIL		SLVKPSFYV YVYSRVKNL		KLFIRQEEV	ILFTRFFYV		TFTYASALW		LMIERFVSL SLAIDAYPL	KLSYGIATV	IVDTVSALV	YLNTLTLAV
A*02 : 03	7									SLVKPSFYV YVYSRVKNL			ILFTRFFYV		ALWEIQQVV		LMIERFVSL	KLSYGIATV		YLNTLTLAV
A*02 : 06	9									SLVKPSFYV YVYSRVKNL			ILFTRFFYV		ALWEIQQVV	NVIPTITQM	LMIERFVSL	KLSYGIATV	IVDTVSALV	YLNTLTLAV
A*11 : 01	6					ASAFFGMSR	KTFPPTEPK							SAFAMMFVK			AIDAYPLTK	KLFAAETLK		TLAVPYNMR
A*24 : 02 /A*23 : 02	9		YYRRATRRI	KMKDLSPRW				SYFIASFRLFA, LFARTRSMW, SFNPETNIL		FYVYSRVKNL	YYQLYSTQL		AYILFTRFF		TYASALWEI					
A*26 : 01	3			DLSPRWYFY		VTPSGTWLTY										NVIPTITQM				
A*03 : 01	13			AGLPYGANK	QLESKMSGK, ALALLLLDR	ASAFFGMSR	AYKTFPPTEPK, KTFPPTEPKK		RIAGHHLGR			QLRARSVSPK		SAFAMMFVK			AIDAYPLTK	KLSYGIATVR, KLFAAETLK		TLAVPYNMR
A*30 : 01	3					VTPSGTWLTY												KLFAAETLK	IVDTVSALVY	
A*31 : 01	5					ASAFFGMSR			AVILRGHLR			RARSVSPKL						KLSYGIATVR		TLAVPYNMR
A*32 : 01	1							RLFARTRSMW												
A*33 : 01	2					ASAFFGMSR			AVILRGHLR											
A*68 : 01	6					ASAFFGMSR			AVILRGHLR HLRIAGHHLGR					FAMMFVKHK				KLSYGIATVR		TLAVPYNMR
A*68 : 02	1															NVIPTITQM	LMIERFVSL			
B*07 : 02	6	LPNNTASWF	PRGQGVPI	SPRWYFYYL								RARSVSPKL				IPTITQMNL	LMIERFVSL			
B*08 : 01	5		FPRGQGVPI						HLRIAGHHL	YVYSRVKNL		KLFIRQEEV		FVKHKHAFL						
B*14 : 02	2	NTASWFTAL	FPRGQGVPI																	
B*15 : 01																				
B*18 : 01	3			SPRWYFYYL	RLNQLESKM			RLFARTRSMW												
B*27 : 05	0																			
B*35 : 01	2		FPRGQGVPI													NVIPTITQM IPTITQMNL				
B*40 : 01	4					MEVTPSGTWL			SELVIGAVIL				AEWFLAYIL						AEIVDTVSAL	
B*44 : 02/03	4					MEVTPSGTWL, PSGTWLTYH			SELVIGAVIL				AEWFLAYILF							
B*51 : 01	4	LPNNTASWF	FPRGQGVPI													IPTITQMNL			CPAEIVDTV	
B*53 : 01	4	LPNNTASWF				APSASAFFGM										IPTITQMNL			CPAEIVDTV	
B*57 : 01	3			KMKDLSPRW		APSASAFFGM		LFARTRSMW												
B*58 : 01	1							LFARTRSMW												
CLASS II	19	TASWFTALTQHGKEE, LPNNTASWFTALTQH	NSGPDDQIGYYRRAT QIGYYRRATRRVRGGDGK	MKDLSPRWYFYYLGT LSPRWYFYYLGTGPEAGL SPRWYFYYLGTGPEA	GDAALALLLLDRLNQL LLLLDRLNQLESKMS	SASAFFGMSRIGMEV FGMSRIGMEVTPSGT GMSRIGMEVTPSGTW	VILLNKHIDAYKTFP LLNKHIDAYKTFPPTEPK YKTFPPTEPKKDKKKK		GAVILRGHLRIAGHHLGR	FYVYSRVKNLNSSRV	VLHSYFTSDYYQLY			LCLFLLPSLATVAYF						
**Total**	144	6	8	12	7	17	8	8	9	9	3	5	6	5	5	8	8	9	6	7

**Table 3.** Predicted SARS-CoV-2 epitopes present in the selected peptides. Predicted SARS-CoV-2 epitopes and their HLA restriction present in each peptide based upon the NetMHCpan EL4.1 HLA binding prediction (Reynisson *et al.*, 2020). Positive binding was defined as the top 0.1% scoring epitopes

HLA	No	1	2	3	4	5	6	7	8	9	10	11	12	13	14	15	16	17	18	19
		NP^41-57^	NP^65-94^	NP^100-28^	NP^215-229^	NP^311-335^	NP^350-374^	M^94-111^	M^136-158^	E^50-69^	ORF3^202-19^	ORF7a^76-97^	NSP3^2323-40^	NSP6^3622-647^	NSP8^4082-102^	NSP12^4894-915^	NSP12^5246-63^	NSP13^5455-79^	NSP13^5745-58^	NSP16^6821-45^
A*01 : 01	8		NSSPDDQIGYY	YLGTGPEAGLPY			LLNKHIDAY				HSYFTSDYY YFTSDYYQLY		LVAEWFLAY					ATEETFKLSY	IVDTVSALVY	
A*02 : 01	4				LLLDRLNQL									FLLPSLATV	SALWEIQQVV ALWEIQQVV					
A*02 : 03	2													FLLPSLATV						TLAVPYNMRV
A*02 : 06	2													FLLPSLATV	SALWEIQQV					
A*11 : 01	4	FTALTQHGK		AGLPYGANK			AYKTFPPTEPK					SVSPKLFIR								
A*24 : 02 /A*23 : 02	7			YYLGTGPEAGL						LVKPSFYVY	SYFTSDYYQL		EWFLAYILF	MFVKHKHAF						VPYNMRVIHF, KYTQLCQYL
A*26 : 01	2												LVAEWFLAY						EIVDTVSAL	
A*03 : 01	2	FTALTQHGK								LVKPSFYVY										
A*30 : 01	1									SFYVYSRVK										
A*31 : 01	2									SLVKPSFYVY			LVAEWFLAY							
A*32 : 01	3								RIAGHHLGR	RVKNLNSSR		RSVSPKLFIR								
A*33 : 01	3								LVIGAVILR				FLAYILFTR			DALFAYTKR				
A*68 : 01	9	FTALTQHGK		EAGLPYGANK			YKTFPPTEPK		ELVIGAVILR			SVSPKLFIR	FLAYILFTR			DALFAYTKR		LSYGIATVR		LTLAVPYNMR
A*68 : 02	4														NTFTYASAL		LMIERFVSL	ETFKLSYGI	EIVDTVSAL	
B*07 : 02	0																			
B*08 : 01	5			DLSPRWYF	LLLDRLNQL									MMFVKHKHA, MMFVKHKHAF		FAYTKRNVI				
B*14 : 02	0																			
B*15 : 01	9			DLSPRWYFYY, KMKDLSPRWY			LLNKHIDAY			LVKPSFYVY	VLHSYFTSDY	RQEEVQELY	LVAEWFLAY	MMFVKHKHAF				TLKATEETF		
B*18 : 01	1			KDLSPRWYF																
B*27 : 05	1									SRVKNLNSSR										
B*35 : 01	6		SPDDQIGYY	GPEAGLPY		TPSGTWLTY							LVAEWFLAY		TCDGNTFTY		FVSLAIDAY			
B*40 : 01	2			KDLSPRWYF															AEIVDTVSAL	
B*44 : 02/03	4			KDLSPRWYF		MEVTPSGTW												ATEETFKLSY, TEETFKLSY		
B*51 : 01	5				DAALALLLL	SAFFGMSR									SALWEIQQV	FAYTKRNVI				LAVPYNMRV
B*53 : 01	0																			
B*57 : 01	4											RARSVSPKLF	LAYILFTRF		NTFTYASALW					LTLAVPYNM
B*58 : 01	3		NSSPDDQIGY												NTFTYASALW					LTLAVPYNM
CLASS II	2							SFRLFARTRSMWSF						LCLFLLPSLATVAYF						
**Total**	95	3	3	11	3	3	4	1	3	7	4	5	9	8	8	4	2	6	4	6

Significant variation exists between bat coronaviruses related to SARS-CoV-2 and between other coronaviruses known to infect humans. Conservation of the 20 SARS-CoV-2 peptides with 93 other coronaviruses was assessed (Table 4). High sequence conservation between SARS-CoV-2 and other Serbecoviruses, including SARS-CoV-1 and bat derived SARS-like viruses, was demonstrated. Peptides derived from ORF1ab demonstrated greater conservation between viruses compared to peptides derived from the structural proteins such as the nucleoprotein. High conservation was also observed within the peptides between SARS-CoV-2 and Marbecoviruses, including MERS-CoV, responsible for pathogenic human infection. Again, higher levels of conservation were observed for the ORF1ab peptides. Some conservation was seen for coronaviruses more distantly related to SARS-CoV-2 such as Embecovirus, Duvinacovirus and Setracovirus genera including the viruses OC43, NL63, 229E, HKU1 which infect humans, consistent with recent studies detecting T-cell responses against SARS-CoV-2 in uninfected individuals [13–17]. The conservation in the peptides between SARS-CoV-2 and 93 other coronaviruses was then compared to conservation within regions of the Spike protein known to be targets for neutralising antibody (Table 4). The receptor binding domain (RBD) and the N-terminal domain (NTD) of the spike protein demonstrated greater variation between SARS-CoV-2 and the 93 other coronaviruses relative to the SARS-CoV-2 peptides. For example, the majority of Serbecoviruses demonstrated 100% homology with SARS-CoV-2 in the NSP16^6821-452^ peptide. In contrast the same viruses demonstrated approximately 47% homology to the SARS-CoV-2 NTD and between 60–70% to the SARS-CoV-2 RBD. These data indicate that immune responses raised against the SARS-CoV-2 peptides identified here could mediate cross immunity against diverse coronavirus strains, including those containing spike proteins with limited homology to SARS-CoV-2.

**Table 4.** Conservation of the candidate peptides and regions of the Spike protein between different Coronaviruses. Amino acid conservation was assessed for the peptides selected from the membrane, envelop, ORF3a, ORF7a, nucleoprotein and non-structural proteins between SARS-CoV-2 reference strain (YP_009724389.1) and 94 other coronaviruses. Conservation between reference strain (YP_009724389.1) and 94 other coronaviruses for the receptor binding domain (RBD) and N-terminal, domain (NTD) both antigenic sites for neutralising antibody, was also performed

Genus		Strain	ORF7a	ORF3a	Envelope	Membrane		Nucleocapsid						ORF1ab									Spike			
	Subgenus		ORF7a	OEF3a	E50-69	M94-111	M136-158	NP41-57	NP69-94	NP100-128	NP215-229	NP311-335	NP350-374	NSP32323-45	NSP63622-647	NSP84082-102	NSP124894-915	NSP125246-63	NSP125267-95	NSP13 5455–79	Nsp13(5768–76)	NSP166821-452	NTD	RBD	RBD439-474	RBD550-59
		**SARS-CoV 2 Wuhan-Hu-1**	100.0	100.0	100.0	100.0	100.0	100.0	100.0	100.0	100.0	100.0	100.0	100.0	100.0	100.0	100.0	100.0	100.0	100.0	100.0	100.0	100.0	100.0	100.0	100.0
		**Bat CoV RaTG13**	100.0	100.0	100.0	100.0	100.0	100.0	100.0	100.0	95.7	100.0	100.0	100.0	100.0	100.0	100.0	100.0	100.0	100.0	100.0	100.0	98.5	91.5	81.8	100.0
		**PCoV_GX-P2V**	4.5	77.8	100.0	100.0	100.0	90.0	100.0	100.0	95.7	100.0	100.0	83.3	100.0	90.5	100.0	100.0	100.0	100.0	100.0	100.0	86.7	87.3	81.8	92.0
		**PCoV PCoV_GX-P4L**	100.0	77.8	100.0	100.0	100.0	90.0	100.0	100.0	95.7	100.0	100.0	83.3	100.0	90.5	100.0	100.0	100.0	100.0	100.0	100.0	86.7	87.6	81.8	92.0
		**Bat SARS-like CoV RsSHC014**	100.0	66.7	85.7	100.0	87.0	93.3	95.7	92.9	87.0	96.0	100.0	72.2	85.2	95.2	100.0	100.0	100.0	72.0	100.0	100.0	47.2	75.7	52.3	78.0
		**Bat SARS-like CoV Rs4084**	100.0	66.7	85.7	100.0	87.0	93.3	95.7	92.9	87.0	92.0	100.0	77.8	85.2	95.2	100.0	100.0	100.0	100.0	100.0	100.0	47.2	75.3	52.3	78.0
		**SARS-like CoV WIV16**	90.9	66.7	85.7	100.0	87.0	93.3	95.7	92.9	87.0	96.0	100.0	77.8	85.2	95.2	100.0	100.0	100.0	64.0	100.0	100.0	47.7	74.9	45.5	78.0
		**Bat SARS-like CoV WIV1**	90.9	66.7	85.7	100.0	87.0	93.3	95.7	92.9	87.0	96.0	100.0	77.8	85.2	95.2	100.0	100.0	100.0	72.0	100.0	100.0	47.2	74.9	45.5	78.0
		**Bat SARS-like CoV Rs3367**	100.0	66.7	85.7	100.0	87.0	93.3	95.7	92.9	87.0	96.0	100.0	77.8	85.2	95.2	100.0	100.0	100.0	72.0	100.0	100.0	47.2	74.9	45.5	78.0
		**Bat SARS-like CoV Rs9401**	95.5	66.7	85.7	96.2	87.0	93.3	95.7	92.9	87.0	96.0	100.0	77.8	85.2	95.2	100.0	100.0	100.0	100.0	100.0	100.0	47.2	74.5	45.5	78.0
		**CoV BtRs-BetaCoV/YN2018B**			85.7	100.0	87.0	93.3	95.7	92.9	87.0	96.0	100.0	77.8	85.2	95.2	100.0	100.0	100.0	100.0	100.0	100.0	47.2	74.5	45.5	78.0
		**SARS-related-CoV Civet CoV 007/2004**	0.0	66.7	85.7	96.2	87.0	93.3	95.7	92.9	87.0	96.0	100.0	72.2	85.2	95.2	100.0	100.0	100.0	60.0	100.0	100.0	46.2	73.7	45.5	78.0
		**SARS-related CoV civet010**	0.0	66.7	85.7	96.2	87.0	93.3	95.7	92.9	87.0	96.0	100.0	72.2	85.2	95.2	100.0	100.0	100.0	60.0	100.0	100.0	46.2	73.7	45.5	78.0
		**SARS CoV civet020**	0.0	66.7	85.7	96.2	87.0	93.3	95.7	92.9	87.0	96.0	100.0	72.2	85.2	95.2	100.0	100.0	100.0	60.0	100.0	100.0	46.2	73.7	45.5	78.0
**Betacoronavirus**		**SARS CoV-1 B039**	0.0	66.7	85.7	96.2	87.0	93.3	95.7	92.9	87.0	96.0	100.0	72.2	85.2	95.2	100.0	100.0	100.0	60.0	100.0	100.0	46.2	73.7	45.5	78.0
		**SARS CoV A022**	100.0	66.7	85.7	96.2	87.0	93.3	95.7	92.9	87.0	96.0	100.0	72.2	85.2	95.2	100.0	100.0	100.0	60.0	100.0	100.0	46.2	73.7	45.5	78.0
		**SARS-CoV-1 Tor2**		66.7	85.7	96.2	87.0	93.3	95.7	92.9	87.0	96.0	100.0	72.2	85.2	95.2	100.0	100.0	100.0	100.0	100.0	100.0	46.2	73.4	45.5	78.0
		**SARS-CoV-1 GZ02**	0.0	66.7	9.5	96.2	87.0	93.3	95.7	92.9	87.0	96.0	100.0	72.2	85.2	95.2	100.0	100.0	100.0	60.0	100.0	100.0	45.6	73.0	45.5	76.0
		**SARS-related CoV BtKY72**	100.0	50.0	85.7	92.3	82.6	86.7	91.3	92.9	91.3	92.0	100.0	83.3	77.8	90.5	100.0	100.0	100.0	80.0	92.9	96.0	41.0	73.0	45.5	78.0
		**Bat-CoV BM48-31/BGR/2008**	0.0	55.6	85.7	92.3	87.0	86.7	91.3	92.9	91.3	92.0	100.0	72.2	81.5	95.2	100.0	100.0	100.0	100.0	100.0	96.0	38.5	70.7	31.8	80.0
	**Sarbecovirus**	**Bat CoV BM48-31/BGR/2008**	0.0	55.6	85.7	92.3	87.0	91.3	86.7	92.9	91.3	92.0	80.8	72.2	81.5	95.2	100.0	100.0	100.0	72.0	100.0	96.0	38.5	70.7	31.8	80.0
		**Bat SARS-like CoV-SL-CoVZC45**	0.0	94.4	100.0	100.0	100.0	96.7	95.7	96.4	87.0	96.0	100.0	100.0	100.0	95.2	100.0	100.0	100.0	100.0	100.0	100.0	61.0	69.9	31.8	78.0
		**bat-SL-CoVZXC21**	100.0	100.0	100.0	100.0	100.0	96.7	95.7	96.4	87.0	96.0	100.0	100.0	100.0	95.2	100.0	100.0	100.0	100.0	100.0	100.0	59.0	69.5	29.5	78.0
		**BtRl-BetaCoV/SC2018**			85.7	92.3	87.0	90.0	95.7	92.9	87.0	96.0	92.3	77.8	85.2	95.2	100.0	100.0	100.0	100.0	100.0	100.0	42.1	66.0	31.8	78.0
		**Bat-CoV Rp/Shaanxi2011**		66.7	85.7	96.2	87.0	90.0	95.7	92.9	87.0	96.0	100.0	77.8	85.2	95.2	95.2						41.0	66.0	34.1	82.0
		**CoV BtRs-BetaCoV/YN2018C**			85.7	96.2	87.0	93.3	95.7	92.9	87.0	96.0	100.0	77.8	85.2	95.2	100.0	100.0	100.0	100.0	100.0	100.0	47.2	65.6	31.8	78.0
		**Bat SARS-CoV HKU3-2**	0.0	66.7	85.7	96.2	87.0	90.0	95.7	92.9	87.0	96.0	100.0	72.2	85.2	95.2	100.0	100.0	100.0	56.0	100.0	100.0	47.2	65.3	34.1	80.0
		**Bat SARS CoV HKU3-4**	45.5	5.6	85.7	96.2	87.0	90.0	95.7	92.9	87.0	96.0	100.0	72.2	85.2	95.2	100.0	100.0	100.0	72.0	100.0	100.0	47.2	65.3	34.1	80.0
		**Bat SARS CoV HKU3-3**	90.9	5.6	85.7	96.2	87.0	90.0	95.7	92.9	87.0	96.0	100.0	72.2	85.2	95.2	100.0	100.0	100.0	64.0	100.0	100.0	47.2	65.3	34.1	80.0
		**Bat SARS CoV HKU3-1**	100.0	66.7	85.7	96.2	87.0	90.0	95.7	92.9	87.0	96.0	100.0	72.2	85.2	95.2	100.0	100.0	100.0	64.0	100.0	100.0	47.2	65.3	34.1	80.0
		**BtRs-Beta CoV/GX2013**	100.0	72.2	85.7	96.2	87.0	90.0	95.7	92.9	87.0	96.0	100.0	77.8	85.2	95.2	100.0	100.0	100.0	72.0	100.0	100.0	46.7	65.3	34.1	80.0
		**BtRf-BetaCoV/SX2013**	95.5	61.1	81.0	96.2	87.0	86.7	95.7	92.9	87.0	96.0	100.0	77.8	85.2	95.2	95.5	100.0	100.0	72.0	100.0	100.0	47.7	64.9	34.1	78.0
		**BtRf-BetaCoV/HeB2013**	95.5	61.1	85.7	96.2	87.0	86.7	95.7	92.9	87.0	96.0	100.0	77.8	85.2	95.2	95.5	100.0	100.0	72.0	100.0	100.0	47.7	64.9	34.1	78.0
		**Bat SARS-like CoV YNLF_31C**	100.0	61.1	85.7	96.2	87.0	90.0	95.7	92.9	87.0	96.0	100.0	72.2	85.2	95.2	100.0	100.0	100.0	64.0	100.0	100.0	47.2	64.9	34.1	76.0
		**Bat CoV 279/2005**	0.0	72.2	85.7	96.2	87.0	90.0	95.7	92.9	87.0	92.0	96.2	77.8	85.2	95.2	95.5	100.0	100.0	60.0	100.0	100.0	46.2	64.9	31.8	80.0
		**Bat SARS-like CoV Rs4237**	100.0	66.7	85.7	96.2	87.0	93.3	95.7	92.9	87.0	96.0	100.0	77.8	85.2	95.2	100.0	100.0	100.0	100.0	100.0	100.0	48.2	64.5	31.8	80.0
		**Bat CoV 273/2005**	95.5	61.1	85.7	96.2	87.0	86.7	95.7	92.9	87.0	92.0	100.0	77.8	85.2	95.2	95.5	100.0	100.0	56.0	100.0	100.0	48.2	64.1	31.8	78.0
		**Bat-CoV Cp/Yunnan2011**		66.7	85.7	100.0	87.0	90.0	95.7	92.9	87.0	96.0	100.0	83.3	85.2	95.2	95.2						47.7	64.1	34.1	80.0
		**SARS CoV Rs_672/2006**	100.0	66.7	85.7	96.2	87.0	90.0	95.7	92.9	87.0	96.0	100.0	72.2	85.2	95.2	100.0	100.0	100.0	72.0	100.0	100.0	43.6	64.1	29.5	80.0
		**BtRs-Beta-CoV/YN2018D**			85.7	100.0	87.0	93.3	95.7	92.9	87.0	96.0	100.0	77.8	85.2	95.2	100.0	100.0	100.0	100.0	100.0	100.0	43.6	64.1	29.5	82.0
		**BtRs-BetaCoV/YN2013**	100.0	61.1	85.7	96.2	87.0	95.7	93.3	92.9	87.0	96.0	80.8	72.2	85.2	95.2	100.0	100.0	100.0	72.0	100.0	100.0	46.7	63.3	31.8	82.0
		**BtRf-Beta-CoV/JL2012**	95.5	55.6	81.0	96.2	87.0	86.7	95.7	92.9	87.0	96.0	100.0	77.8	85.2	95.2	95.5	100.0	100.0	72.0	100.0	100.0	35.9	63.3	34.1	78.0
		**MERS-related CoV H.savii/Italy/206645-40/2011**		0.0	23.8	73.1	34.8	60.0	60.9	67.9	47.8	40.0	46.2	38.9	51.9	23.8	86.4	94.4	65.5	100.0	85.7	80.0	16.4	24.7	13.6	26.0
		**MERS-related CoV P.khulii/Italy/206645-63/2011**		0.0	23.8	73.1	34.8	60.0	60.9	67.9	47.8	40.0	46.2	38.9	51.9	23.8	86.4	94.4	65.5	100.0	85.7	80.0	16.4	24.7	13.6	26.0
		**Pipistrellus bat CoV HKU5**		16.7	9.5	69.2	34.8	60.0	65.2	64.3	30.4	32.0	38.5	50.0	51.9	28.6	86.4	94.4	65.5	100.0	85.7	80.0	16.9	23.6	13.6	26.0
		**MERS-CoV NL140422**		5.6	28.6	76.9	39.1	60.0	60.9	64.3	34.8	36.0	46.2	27.8	55.6	28.6	86.4	94.4	65.5	100.0	85.7	80.0	16.4	23.6	11.4	20.0
		**MERS-related CoV KOR/KCDC/001_2018-TSVi**		5.6	23.8	73.1	39.1	60.0	65.2	67.9	47.8	36.0	50.0	38.9	55.6	28.6	81.8	94.4	65.5	100.0	85.7	88.0	16.4	23.2	13.6	20.0
		**Hu beta CoV 2 c EMC/2012**		5.6	23.8	73.1	39.1	60.0	65.2	67.9	47.8	36.0	50.0	38.9	55.6	28.6	86.4	94.4	65.5	72.0	85.7	88.0	16.4	23.2	13.6	20.0
		**MERS-related CoV MA_30**		5.6	23.8	73.1	39.1	60.0	65.2	67.9	47.8	36.0	50.0	38.9	55.6	28.6	86.4	94.4	65.5	100.0	85.7	88.0	16.4	23.2	13.6	20.0
		**BetaCoV England 1**		5.6	23.8	73.1	39.1	60.0	65.2	67.9	47.8	36.0	50.0	38.9	55.6	28.6	86.4	94.4	65.5	100.0	85.7	88.0	16.4	23.2	13.6	20.0
	**Merbecovirus**	**BtCoV/133/2005**		0.0	28.6	65.4	43.5	56.7	65.2	60.7	39.1	36.0	46.2	50.0	51.9	19.0	86.4	83.3	65.5	56.0	85.7	88.0	14.9	23.2	13.6	24.0
		**MERS-related CoV BtVs-BetaCoV/SC2013**		5.6	28.6	73.1	39.1	60.0	65.2	64.3	39.1	40.0	42.3	38.9	51.9	28.6	90.9	94.4	65.5	72.0	85.7	76.0	12.8	23.2	13.6	22.0
		**Tylonycteris bat CoV HKU4 BtTp-BetaCoV/GX2012**		0.0	28.6	65.4	43.5	56.5	56.7	60.7	43.5	36.0	23.1	44.4	51.9	19.0	86.4	83.3	65.5	64.0	85.7	88.0	15.9	22.8	13.6	24.0
		**Hypsugo bat CoV HKU25**			28.6	65.4	43.5	56.7	65.2	64.3	39.1	36.0	46.2	38.9	51.9	33.3	86.4	94.4	65.5	100.0	85.7	80.0	14.9	22.8	11.4	20.0
		**Bat-CoV HKU4-1**	9.1	0.0	28.6	65.4	43.5	56.7	65.2	60.7	39.1	36.0	46.2	50.0	48.1	23.8	86.4	83.3	65.5	60.0	85.7	88.0	15.9	22.4	11.4	24.0
		**Tylonycteris bat CoV HKU4**			28.6	65.4	43.5	56.7	65.2	60.7	39.1	36.0	46.2	50.0	48.1	23.8	86.4	83.3	65.5	100.0	85.7	88.0	15.9	22.4	11.4	24.0
		**CoV Neoromicia/PML-PHE1/RSA/2011**		5.6	23.8	73.1	34.8	56.7	60.9	67.9	47.8	40.0	46.2	44.4	51.9	28.6	86.4	94.4	65.5	72.0	85.7	84.0	14.9	21.2	20.5	26.0
		**BetaCoV Erinaceus/VMC/DEU/2012**		5.6	28.6	73.1	34.8	56.7	56.5	64.3	39.1	36.0	38.5	27.8	55.6	28.6	86.4	94.4	65.5	100.0	85.7	76.0	12.3	20.5	18.2	24.0
		**Myotis ricketti alpha-CoV Sax-2011**		11.1	14.3	61.5	21.7	23.3	17.4	32.1	21.7	12.0	11.5	5.6	44.4	28.6	68.2	72.2	31.0	72.0	85.7	72.0	10.8	9.7	6.8	22.0
		**Beta CoV HKU24**			33.3	69.2	39.1	36.7	26.1	57.1	26.1	28.0	19.2	44.4	48.1	28.6	77.3	83.3	48.3	100.0	92.9	84.0	12.8	24.7	11.4	24.0
	**Embecovirus**	**Rabbit CoV HKU14**			28.6	69.2	39.1	36.7	26.1	57.1	21.7	32.0	19.2	38.9	40.7	33.3	77.3	83.3	48.3	100.0	92.9	84.0	10.3	22.8	6.8	24.0
		**Hu-CoV HKU1**			38.1	69.2	39.1	30.4	40.0	60.7	21.7	24.0	19.2	38.9	40.7	38.1	72.7	83.3	48.3	72.0	85.7	84.0	11.8	21.2	6.8	26.0
		**Hu-CoV OC43**			85.7	69.2	39.1	36.7	26.1	57.1	21.7	32.0	23.1	38.9	40.7	33.3	77.3	83.3	48.3	60.0	92.9	84.0	10.3	20.8	6.8	24.0
		**Rousettus bat CoV GCCDC1**	100.0	16.7	28.6	73.1	39.1	36.7	47.8	50.0	34.8	36.0	50.0	33.3	48.1	38.1	86.4	88.9	69.0	100.0	92.9	80.0	15.4	24.3	11.4	26.0
	**Nobecovirus**	**Rousettus bat CoV HKU9**	45.5	22.2	23.8	69.2	39.1	40.0	43.5	46.4	30.4	28.0	65.4	33.3	37.0	38.1	90.9	88.9	69.0	100.0	92.9	80.0	15.9	22.4	11.4	24.0
		**Bat CoV HKU9-5-1**		16.7	23.8	69.2	39.1	50.0	47.8	46.4	26.1	24.0	65.4	38.9	40.7	38.1	90.9	88.9	69.0	72.0	92.9	84.0	16.4	22.0	11.4	28.0
		**CoV BtRs-BetaCoV/YN2018A**			85.7	96.2	87.0	90.0	95.7	92.9	87.0	96.0	100.0	72.2	85.2	95.2	100.0	100.0	100.0	100.0	100.0	100.0	48.2	64.9	31.8	82.0
		**Bat CoV Jiyuan-84**			85.7	96.2	87.0	86.7	95.7	92.9	87.0	96.0	100.0	77.8	85.2	95.2	95.5	100.0	100.0	100.0	100.0	100.0	47.7	64.9	34.1	78.0
		**Bat CoV JTMC15**	40.9	55.6	81.0	96.2	87.0	86.7	95.7	92.9	87.0	96.0	100.0	77.8	85.2	95.2	95.5	100.0	100.0	80.0	100.0	100.0	36.4	63.3	34.1	78.0
	**Unclassified**	**Apodemus peninsulae CoV**			33.3	69.2	39.1	40.0	26.1	57.1	26.1	28.0	19.2	44.4	48.1	28.6	77.3	83.3	48.3	100.0	92.9	84.0	12.8	23.9	11.4	24.0
		**Bat Vs-CoV-1**		5.6	28.6	73.1	39.1	60.0	65.2	64.3	39.1	40.0	42.3	38.9	51.9	28.6	90.9	94.4	65.5	72.0	85.7	76.0	16.4	22.4	9.1	26.0
		**Bat-CoV CMR704-P12**		16.7	19.0	61.5	30.4	40.0	43.5	53.6	30.4	32.0	65.4	38.9	44.4	28.6	90.9	88.9	69.0	96.0	85.7	80.0	13.3	22.4	11.4	18.0
		**MERS-related CoV BtVs-BetaCoV/SC2013**		5.6	23.8	73.1	34.8	56.7	60.9	67.9	47.8	40.0	46.2	44.4	51.9	28.6	86.4	94.4	65.5	100.0	85.7	84.0	14.9	21.2	20.5	26.0
		**Bat coronavirus PDF-2180**		5.6	23.8	73.1	34.8	60.0	65.2	67.9	43.5	40.0	42.3	44.4	51.9	28.6	86.4	94.4	65.5	100.0	85.7	84.0	14.9	19.7	18.2	28.0
		**BtRf-AlphaCoV/HuB2013**	13.6	11.1	14.3	53.8	21.7	30.0	8.7	46.4	8.7	16.0	7.7	16.7	40.7	19.0	77.3	77.8	31.0	60.0	78.6	68.0	11.3	10.0	4.5	18.0
	**Decacovirus**	**bat-CoV HKU10**	100.0	16.7	19.0	53.8	21.7	26.7	8.7	42.9	13.0	8.0	7.7	22.2	48.1	19.0	77.3	72.2	31.0	72.0	78.6	76.0	9.2	9.3	4.5	14.0
		**Rousettus bat CoV HKU10**	4.5	16.7	19.0	53.8	21.7	26.7	8.7	42.9	13.0	8.0	7.7	22.2	48.1	19.0	77.3	72.2	31.0	100.0	78.6	76.0	13.3	8.5	2.3	16.0
	**Duvinacovirus**	**Hu-CoV 229E**			14.3	53.8	30.4	30.0	8.7	46.4	13.0	28.0	3.8	5.6	48.1	23.8	68.2	72.2	27.6	60.0	85.7	72.0	4.1	9.7	6.8	26.0
		**Human CoV 229E**			14.3	53.8	30.4	26.7	8.7	46.4	13.0	32.0	3.8	5.6	51.9	23.8	72.7	72.2	27.6	72.0	85.7	76.0	4.1	8.5	4.5	26.0
	**Setracovirus**	**NL63-related bat CoV BtKYNL63-9b**		5.6	19.0	65.4	26.1	30.0	8.7	39.3	13.0	28.0	19.2	5.6	48.1	23.8	81.8	72.2	31.0	96.0	85.7	72.0	6.7	9.7	4.5	18.0
		**Hu-CoV NL63**		5.6	9.5	53.8	21.7	33.3	8.7	42.9	17.4	24.0	7.7	5.6	48.1	19.0	81.8	72.2	27.6	60.0	85.7	72.0	9.2	9.3	13.6	16.0
	**Nyctacovirus**	**Bat-CoV/P.kuhlii**		16.7	14.3	50.0	21.7	26.7	4.3	57.1	13.0	20.0	11.5	22.2	44.4	19.0	68.2	72.2	31.0	96.0	85.7	72.0	7.2	11.2	4.5	22.0
		**BtNv-AlphaCoV/SC2013**		16.7	14.3	50.0	21.7	30.0	4.3	50.0	13.0	28.0	19.2	16.7	37.0	19.0	72.7	72.2	31.0	100.0	85.7	72.0	6.2	8.9	2.3	16.0
**Alphacoronavirus**	**Myotacovirus**	**Pipistrellus bat CoV HKU5 BtPa-BetaCoV/GD2013**		5.6	9.5	69.2	34.8	60.0	65.2	64.3	30.4	32.0	38.5	50.0	51.9	28.6	86.4	94.4	65.5	72.0	85.7	80.0	17.4	22.8	13.6	26.0
		**BtMr-AlphaCoV/SAX2011**		11.1	14.3	61.5	21.7	23.3	17.4	32.1	21.7	12.0	11.5	5.6	44.4	28.6	68.2	72.2	31.0	100.0	85.7	72.0	10.8	9.7	6.8	22.0
		**Bat CoV CDPHE15/USA/2006**		33.3	14.3	46.2	17.4	30.0	13.0	39.3	21.7	16.0	11.5	16.7	40.7	14.3	68.2	72.2	31.0	100.0	85.7	64.0	8.7	6.6	2.3	20.0
	**Rhinacovirus**	**bat-CoV HKU2**	9.1		14.3	53.8	26.1	36.7	13.0	46.4	8.7	20.0	7.7	11.1	48.1	28.6	72.7	72.2	31.0	60.0	78.6	72.0	8.2	3.1	9.1	20.0
		**BtRf-AlphaCoV/YN2012**		11.1	14.3	53.8	26.1	30.0	13.0	46.4	8.7	20.0	7.7	11.1	48.1	28.6	72.7	72.2	31.0	100.0	78.6	72.0	7.2	2.7	4.5	20.0
	**Pedacovirus**	**bat-CoV 512**		22.2	23.8	53.8	17.4	30.0	13.0	42.9	26.1	24.0	19.2	16.7	55.6	14.3	77.3	72.2	31.0	60.0	78.6	68.0	9.2	8.1	2.3	22.0
		**Miniopterus bat-CoV 1 AFCD62**	100.0	11.1	14.3	61.5	21.7	33.3	13.0	50.0	17.4	20.0	19.2	16.7	37.0	19.0	72.7	72.2	27.6	72.0	85.7	64.0	8.7	11.2	9.1	22.0
	**Minunacovirus**	**BtMf-AlphaCoV/AH2011**		11.1	14.3	61.5	21.7	33.3	8.7	50.0	17.4	20.0	19.2	16.7	37.0	19.0	72.7	72.2	27.6	72.0	85.7	64.0	7.7	11.2	9.1	22.0
		**Bat CoV 1B**		11.1	9.5	61.5	21.7	33.3	13.0	50.0	17.4	20.0	15.4	16.7	37.0	19.0	72.7	72.2	27.6	72.0	85.7	64.0	8.7	10.8	6.8	22.0

Most HLA class I restricted epitopes consist of 8-10mer amino acid sequences. Whilst high levels of conservation in the peptides was demonstrated between Coronaviruses (Table 4) a relatively small amount of variation can significantly alter recognition by either T-cell or antibody-based immune responses, demonstrated by observations that amino acid substations allow immune escape from neutralising antibody, however variation within one or two amino acids within the epitope sequence may still allow for T-cell recognition, albeit sometimes with altered TCR avidity for the peptide/MHC complex. This is particularly true for conservative amino acid substation such as isoleucine and leucine. For this reason, the conservation of the 125 validated, HLA-class I restricted epitopes identified in SARS-CoV-2 peptides were determined in each of the 20 peptides from each of the 93 coronaviruses used previously (Table 5). All 93 viruses had ten or more epitopes with homology to the SARS-CoV-2 epitopes within the peptides, including at least one identical epitope. Merbecoviruses contained between 16–35 epitopes, including between 1–11 identical epitopes. There was a high degree of epitope conservation within the Serbecoviruses most closely related to SARS-CoV-2. These data add further support for the potential cross reactivity of immune responses to the peptides between diverse coronaviruses.

**Table 5.**
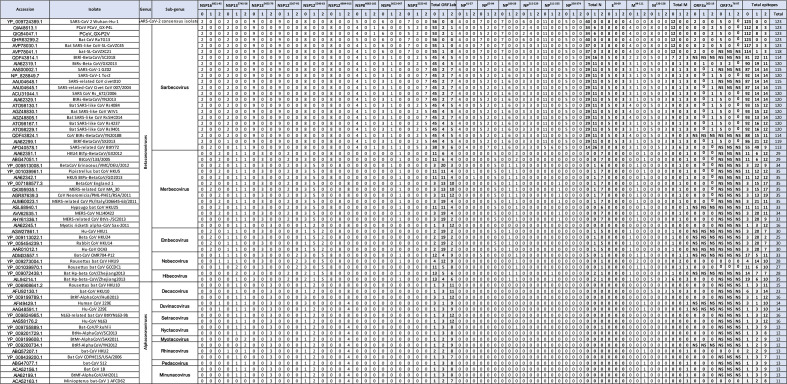
Conservation of epitopes between SARS-CoV-2 and other coronaviruses. Experimentally validated epitopes deposited in IEDB and present within the 21 peptides chosen in this study were identified and their presence and variability assessed in 93 different coronaviruses. The number of identical epitopes or those harbouring one or two amino acid substations was determined.

### Selection and T-cell reactivity of synthetic long peptides from SARS-CoV-2

Next, each peptide region was assessed for its the suitability to synthesize as synthetic long peptides (SLP) since the physiochemical properties of the peptides may make them unsuitable for synthesis or for targeting toward antigen presenting cells or homology between the peptides and human proteins may make them unsuitable for vaccination. Amino acid modifications were made outside of epitope containing regions in order to improve synthesis, stability and internalisation. Each of the peptides was differently conserved between other coronaviruses and contained a different number of epitopes restricted to different HLA. A total of 16 of these candidate SLP were selected as an immunogenic pool for *in vitro* assessment. These peptides are all water soluble and positively charged, which aids their internalisation into antigen presenting cells. The sequence length of between 21–30 amino acids allows for the presence of negatively charged, or hydrophobic amino acids, and the epitopes containing them, without resulting in an overall negative charge or solubility of the peptides.

**Fig. 1.**
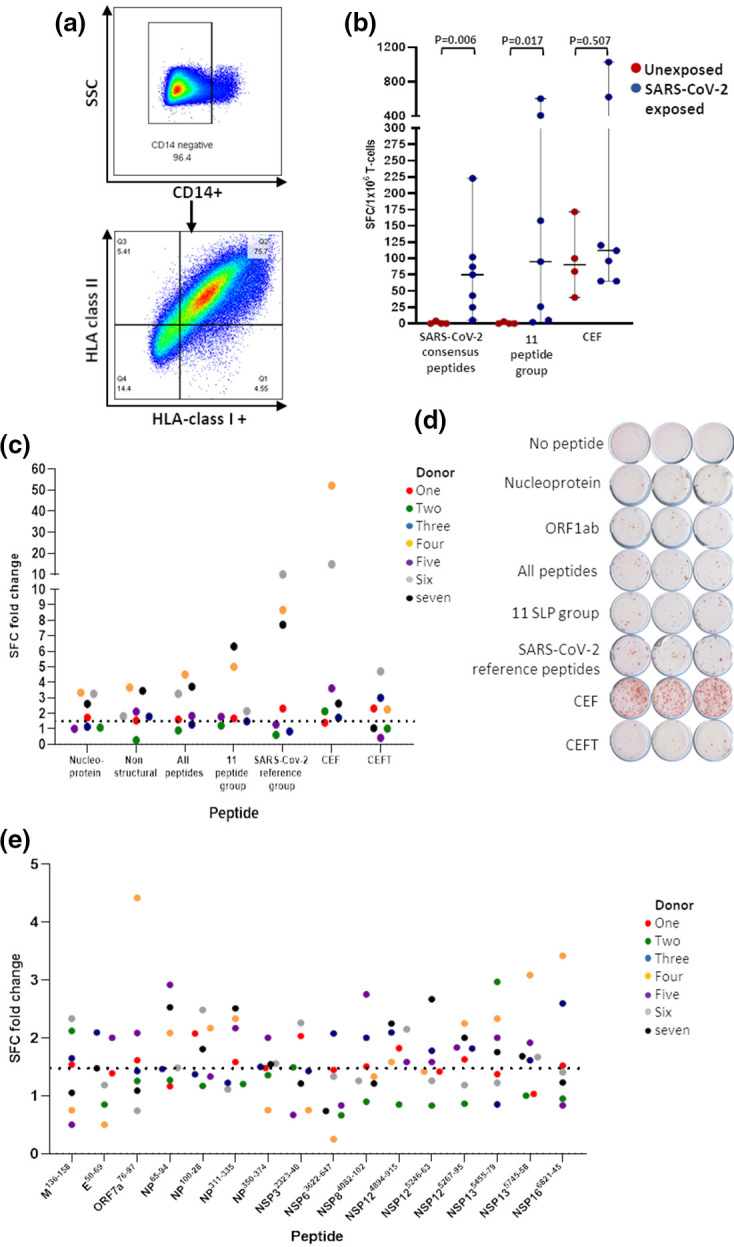
IFN-γ responses to the SARS-CoV-2 synthetic long peptides. PBMC from SARS-CoV-2 seronegative and seropositive donors were used to generate monocyte derived dendritic cells (mDDC) (**a**) and incubated with SARS-CoV-2 consensus peptides, a group of 11 SLP or CEF peptides prior to co culture with autologous T-cells in triplicate wells of ELISPOT plates to measure T-cell responses (**b**). MDDC from seven SARS-CoV-2 seropositive individuals were loaded with groups of SLP, SARS-CoV-2 consensus peptides or CEF and CEFT positive controls peptide pools and incubated with autologous T-cells for 48 h in triplicate wells of IFN-γ ELISPOT plates (**c**) Example of ELISPOT responses to SARS and positive control peptide pools (**d**). MDDC from the seropositive donors were also loaded with Individual SLP and co cultured with autologous T-cells in order to measure T-cell responses to each peptide (**e**). Positive responses were defined as a fold increase of 1.5 and statistical significance using a Student’s *t*-test.

The selected peptides were assessed for their ability to activate T-cell responses. PBMC from seven individuals with previous SARS-CoV-2 infection and four individuals seronegative for SARS-CoV-2, without selection for specific HLA expression, were used to generate monocyte derived dendritic cells (Fig. 1a), loaded with peptides and combined in IFN-γ ELISPOT plates with autologous T-cells (Fig. 1b–e). The SARS-CoV-2 peptides were assessed as peptide groups including a nucleoprotein group, ORF1ab group and an 11-peptide group which combined peptides from different regions based upon their expression of class II epitopes and the most highly conserved class I epitopes (identified in Table 5; details in Table S2). These were compared to a SARS-CoV-2 reference group of overlapping peptides to the Spike, nucleoprotein and membrane protein and to CEF and CEFT positive control peptides. The SAR-CoV-2 groups induced IFN-γ from mDDC-T-cell co-cultures derived from individuals with previous SARS-CoV-2 infection but not seronegative individuals (Fig. 1c, d). Each peptide group induced the expression of IFN-γ from the DC-T-cell co-cultures of between 3–5 donor PBMC (Fig. 1c) providing support for the feasibility of grouping the peptides. The 16 SARS-CoV-2 were individually tested and induced IFN-γ expression from the T-cells of between 1–5 individuals with previous SARS-CoV-2 infection (Fig. 1e, Table S2) but none against the T-cells from seronegative donors (data not shown). IFN-γ responses were observed against between 2–9 peptides (average of five) derived from different between 2–6 viral proteins (average of 3.7) from each of the seven donors.

**Fig. 2.**
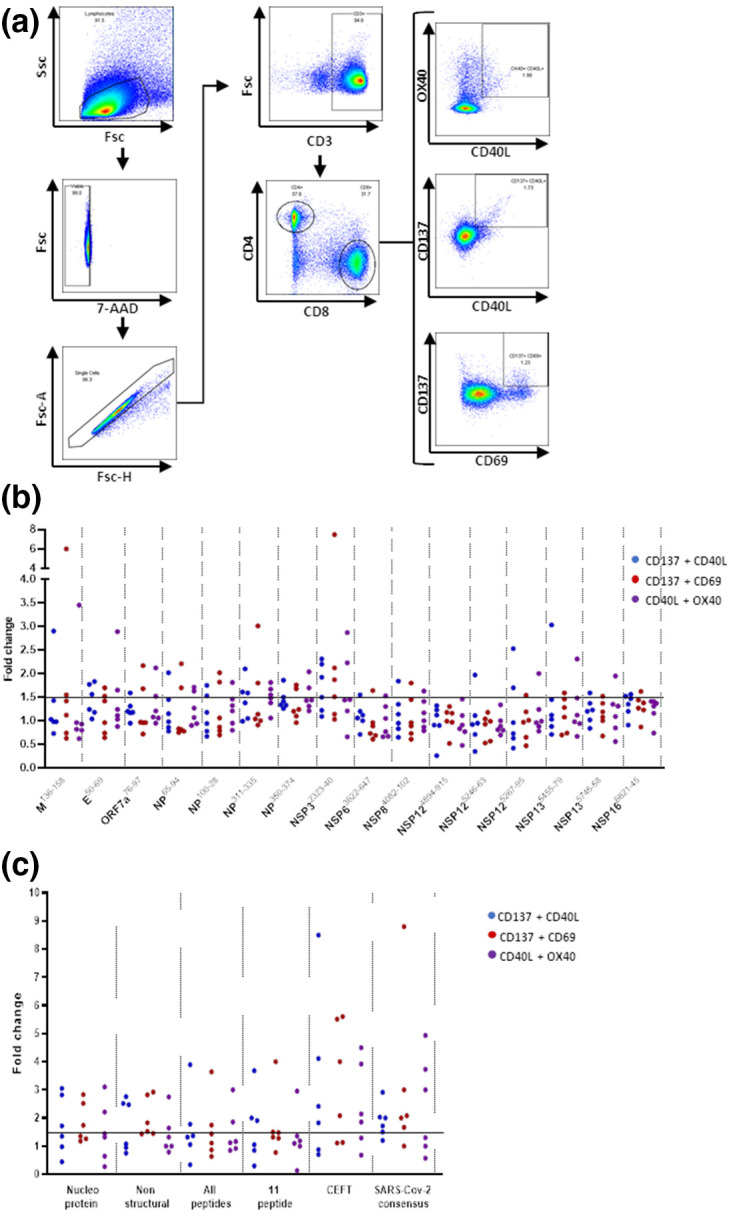
CD4+ T cell responses to SARS-CoV-2 derived synthetic long peptides. Peptide loaded MDDC and T-cell co-cultures derived from six seropositive donors were assessed by flow cytometry for the expression of activation induced markers CD137, CD40L and OX40 (**a**). Individual SARS-CoV-2 derived peptides increase the expression of activation induced markers from CD4+T cells (**b**). Groups of SARS-CoV-2 derived or a class II restricted peptide pool increase the expression of activation induced markers from CD4+T cells. Positive responses were defined as a >1.5-fold increase over the no peptide controls.

**Fig. 3.**
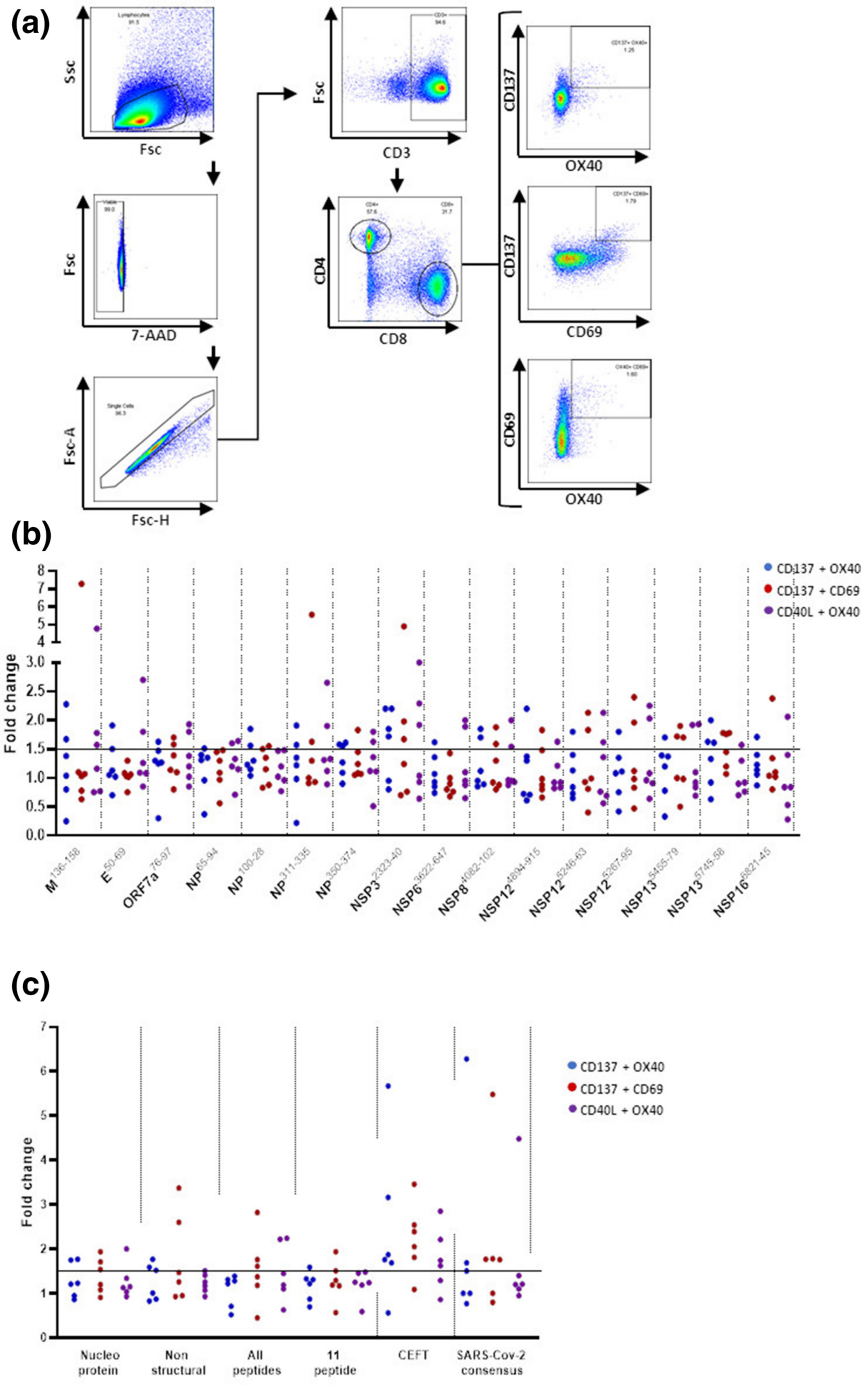
CD8+T cell responses to SARS-CoV-2 derived peptides. Peptide loaded MDDC and T-cell co-cultures from six seropositive donors were assessed by flow cytometry for the expression of activation induced markers CD137, CD69 and OX40 (**a**). Individual SARS-CoV-2 derived peptides increase the expression of activation induced markers from CD8+T cells (**b**). Groups of SARS-CoV-2 derived or a class II restricted peptide pool increase the expression of activation induced markers from CD8+T cells. Positive responses were defined as a >1.5-fold increase over the no peptide controls.

**Fig. 4.**
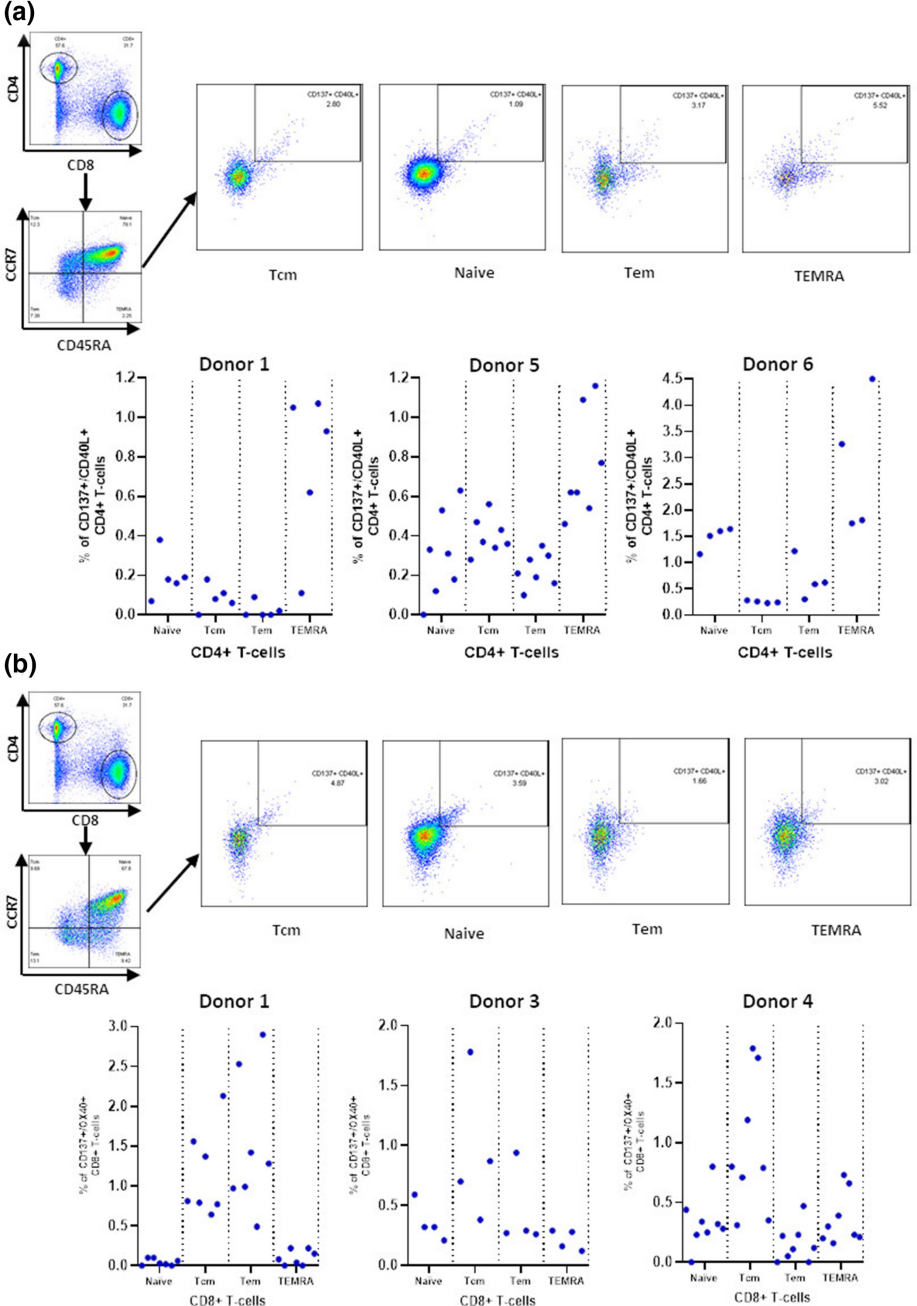
SARS-CoV-2 derived peptides preferentially activate CD4+ and CD8+ T cell subsets. CD4+ T cells subsets were defined based upon their expression of CCR7 and CD45RA and assessed for their expression of activation induced markers from the PBMC of individuals with previous SARS-CoV-2 infection (**a**). Percentage expression of CD137 and CD40L is shown for CD4+T cell subsets from three donors. CD8+ T cells subsets were defined based upon their expression of CCR7 and CD45RA and assessed for their expression of activation induced markers from the PBMC of individuals with previous SARS-CoV-2 infection (**b**). Percentage expression of CD137 and OX40 is shown for CD8+ T cell subsets from three donors.

Next a flow cytometry-based activation induced marker (AIM) assay was used to gain a greater insight into the T-cell responses raised by the peptides (Figs 2 and 3, Table S3). In this assay individuals with prior SARS-COV-2 infection demonstrated responses to each of the peptides through the expression of paired markers including CD137, CD69, CD40L or Ox40. Both CD4+ (Fig. 2a, b) and CD8+ (Fig. 3a, b) T-cell responses indicative of HLA restricted T-cell activation were observed with at least one response observed for each peptide with the exception of NSP^4895-915^ which demonstrated a CD4+ T cell response from the PBMC of one individual but no CD8+ T cell responses. This pattern of responses was consistent with other reports studying anti-SARS T-cell responses [13, 17]. Interestingly the greatest responses were observed amongst CD45RA+CCR7- TEMRA CD4+ T cells (Fig. 4a, b) effector and central memory CD8+ T cells (Fig. 4c, d) consistent with recent studies investigating the phenotype of SARS-CoV-2 reactive T-cells [18, 19].

Taken together these data demonstrate the *in vitro* T-cell antigenicity of SARS-CoV-2 derived SLP containing epitopes restricted to multiple HLA and conserved between SARS-CoV-2 variants and other coronaviruses.

## Discussion

SARS-CoV-2 vaccines based upon the Spike protein have demonstrated between 60–95% efficacy in phase three trials and are now in widespread use. Although these vaccines are highly efficacious numerous issues remain unresolved. These include supply, cost and the requirement of some vaccines for a cold chain. From an immunological perspective there remains concern that variation in the Spike protein may evolve against which antibodies raised by vaccination are less effective, demonstrated to some degree by the Gamma [20] and Delta [21] variants of SARS-CoV-2 in addition to a report detailing extensive but incomplete escape of vaccine elicited neutralization by the Omicron Variant of SARS-CoV-2 . Related issues involve the significant decline of antibody titres within weeks of vaccination in some people [22] and the observation that vaccinated individuals may still become infectious, indicating that regular SARS-CoV-2 vaccination is likely required. The possibility of generating antibody dependent enhancement [23], to novel SARS-CoV-2 variants or other Sarbecoviruses, a phenomenon demonstrated for SARS-CoV-1 [24], and the prospect of future zoonosis with novel Coronaviruses, to which the existing spike-based vaccines may be ineffective, are also of concern.

The broad therapeutic activation of SARS specific T-cell immune responses may resolve or ameliorate a number of these issues. T-cell responses are more stable than humoral responses [15] whilst patients with agammaglobulinemia can recover from COVID-19, supporting a protective role for T-cell immunity [25]. Early induction [26] and antigenic diversity [6] of the SARS-CoV-2-specific T-cell responses is associated with mild COVID-19 and cross reactivity with CD4+ T cell responses to other human coronaviruses are associated with mild infection [13–16]. These observations are consistent with studies showing that a lower frequency [27] and functionality [28, 29] of T-cells is positively correlated with in-hospital death or severity of illness whilst lower counts of total, CD8+, or CD4+ T cells are negatively correlated with patient survival [30]. The Spike protein includes a number of T-cell immunogenic regions but taken together only represents a fraction of the potential anti-SARCoV-2 T-cell response which also targets other SARS-CoV-2 proteins including the nucleoprotein, membrane protein and non-structural proteins of ORF1ab [17, 19, 31–33]. These studies show that significant variation exists in the T-cell antigenic targets of SARS-CoV-2 which may lower the efficacy of spike-based vaccines in patients who demonstrate limited anti-spike T-cell activation. Although infection offers an opportunity to gain immune protection to diverse SARS-CoV-2 antigen, and some studies have identified strong T-cell responses from individuals with asymptomatic or mild COVID-19, other studies suggest that asymptomatic infection may not provide sufficient antigenic stimulation to activate protective, long-lasting anti-SARS-T-cell response [29, 33], supported by observations that CD8+ T cell responses could not be detected in 30% of convalescent individuals [12].

In this study, immunogenic regions from SARS-CoV-2 proteins other than the spike were identified and their conservation amongst selected alpha and beta coronaviruses was assessed. The selected peptides contain multiple epitopes restricted to the most common HLA class I molecules and which have previously demonstrated induction of T-cell activation in response to SARS-CoV-2 (Table 2). Importantly, these peptides were highly conserved between different coronaviruses, particularly of the SARS-like Serbacoviruses, compared to the receptor binding domain of the spike protein, the major antigenic site of neutralising antibodies (Table 4). Each of the peptides induced T-cell responses from the T-cells of at least one individual with previous SARS-CoV-2 infection (Figs 1–3) however future work is warranted in order to extend the limited observations made here. This could define the nature of T-cell responses raised to the peptides in greater detail along with their ability to contribute to protection from SARS-CoV-2 challenge and induce responses from naïve donors, which were not observed in the present study, likely due to the small number of experiments performed using cells from naïve donors. Nethertheless, these data indicate that T-cell responses raised against these peptides may cross react with future SARS-CoV-2 variants, which may evolve to escape neutralising antibody responses, and against future emerging coronaviruses. This is supported by studies screening SARS-CoV-2 epitopes in COVID-19 and uninfected patients which have observed SARS-CoV-2 epitopes specific CD4+ and CD8+ T cell responses in SARS-CoV-2 uninfected individuals, which share homology with epitopes in other human coronaviruses [6, 8–10, 31]. The SARS-CoV-2 peptides studied here include 125 epitopes identified by these epitope screening studies of SARS-CoV-2 patients [6, 8, 9, 19, 31, 32]. A recent study, screening epitopes in 16 COVID-19 patients identified 122 epitopes reactive to T-cells in these individuals [33]. The peptides detailed in the present study share 17 epitopes with this study. Future epitope screening studies may reveal further SARS-CoV-2 specific epitopes which, if part of epitope rich clusters, may identify new regions suitable for the generation of synthetic long peptides of the kind studied here.

The peptides are derived from the immunodominant viral proteins other than the spike (Table 1) so could complement existing spike-based vaccination and contribute to the induction of broad T-cell reactivity associated with improved anti-viral immunity. For example, antibody titres in COVID-19 patients correlate with CD4+ T cell immune responses not just to the spike protein, but also to the nucleoprotein and membrane protein [14, 34] and the peptides studied here include multiple class II restricted epitopes from the nucleoprotein and membrane protein not present in existing spike-based vaccines. Harnessing CD4+ T cell epitopes from other SARS-CoV-2 antigen represents a strategy for improving the response or longevity of protection afforded by existing spike-based vaccines, particularly given observations that the diversity [6], functionality [18, 35] and quality [36] of CD4+ T cell activation supports the generation of cellular and humoral immune responses associated with protection. These observations are supported by the reports that hospitalised patients with robust B-cell responses yet suffering from severe COVID-19 infection demonstrate limited activation of circulating CD4+ follicular T-cells, indicative of the importance of these cells to effective humoral immunity [37]. Vaccine approaches including the envelop and nucleoprotein are under investigation [38, 39], consistent with this approach.

A recent study showed that CD8+ T cell responses against SARS-CoV-2 were raised against approximately 17 epitopes derived from between 1–6 viral proteins (average 2.7) [17]. In our *in vitro* experiments DC-T-cell co-cultures generated from the PBMC of individuals with previous SARS-CoV-2 infection responded with IFN-γ expression to an average of five peptides derived from an average of four proteins. Previous studies have demonstrated that broad T-cell responses against multiple epitopes are more effective than narrow responses targeting fewer epitopes [40–42] and less susceptible to exhaustion [43] indicating that broadening the anti-SARS-CoV-2 T-cell response from vaccination is desirable.

Analysis of T-cell responses to the ChAdOx1 spike-based vaccine showed that nearly 30% of unique TCRs raised by the vaccine mapped to a single region of the spike protein which is mutated in the Beta variant of SARS-CoV-2 [44]. This may contribute to the failure of ChAdOx1 to protect against mild-to-moderate COVID-19 [20]. These studies suggest that variation in SARS-CoV-2 has the potential to reduce vaccine efficacy and support the use of SARS-CoV-2 antigen derived from non-spike proteins.

Synthetic long peptides of the kind studied here have been used in numerous therapeutic vaccines for both infectious disease and cancer and demonstrated an ability to induce efficacious T-cell responses [45–47]. Peptide based vaccines are inherently safe, can be easily manufactured, combined with different adjuvants, including those selected for therapeutic properties such as trained innate immunity, and do not have the same requirements for cold chains as other vaccines. They may be useful alternatives to other vaccine designs for the generation of broad T-cell responses since they exclude non-immunogenic regions and avoid the generation of non-neutralising antibody responses which may be induced by whole virus vaccines [48] and are associated with ADE or toxicity. Alternatively, the peptide regions identified here could also be incorporated into mRNA-based vaccines. Currently vaccination with whole, killed SARS-CoV-2 virions, which have the potential to induce T-cell responses against each viral protein, have demonstrated lower efficacy compared to mRNA or viral vector-based vaccines indicating that other methods of broadening the antigenic repertoire of SARS-CoV-2 vaccines are needed. The peptides studied here are candidate SARS-CoV-2 immunogens with the potential to increase the breadth and cross reactivity of T-cell activation to existing SARS-CoV-2 vaccines.

## Supplementary Data

Supplementary material 1
